# Roxadustat-Induced Central Hypothyroidism in Renal Anemia Patients: Insights From Antineutrophil Cytoplasmic Antibody-Related Vasculitis Cases

**DOI:** 10.7759/cureus.61843

**Published:** 2024-06-06

**Authors:** Daichi Omote, Nobuhiko Kuramoto, Atsuko Tomikawa, Masahiro Yasui, Masashi Fukuta, Masaki Hiraguri

**Affiliations:** 1 Nephrology, Narita Red Cross Hospital, Narita-shi, JPN; 2 Rheumatology, Narita Red Cross Hospital, Narita-shi, JPN

**Keywords:** anca-related vasculitis, renal anemia, central hypothyroidism, roxadustat, hif-ph inhibitors

## Abstract

The introduction of hypoxia-inducible factor-prolyl hydroxylase (HIF-PH) inhibitors in Japan in 2019 for treating renal anemia in hemodialysis patients has resulted in an adverse event: central hypothyroidism. Although this adverse event was not widely recognized by the public, it was first documented in Japan in 2021. Despite limited case reports on roxadustat, an oral HIF-PH inhibitor that induces central hypothyroidism, this condition typically improves rapidly upon discontinuation of the drug. In this report, we present rare cases of roxadustat-induced central hypothyroidism in two patients: a woman in her 80s and a man in his 60s, neither of whom had prior thyroid disease. Both patients developed central hypothyroidism shortly after starting roxadustat treatment for renal anemia associated with antineutrophil cytoplasmic antibody-related vasculitis. Notably, neither patient had pituitary tumors or other pituitary hormone disorders. Thyroid function improved with levothyroxine treatment, even when oral roxadustat was continued. Roxadustat may induce central hypothyroidism, highlighting the importance of regularly measuring and evaluating thyroid function when administering this drug to monitor possible changes in thyroid hormone levels.

## Introduction

Hypoxia-inducible factor (HIF) is a pivotal transcription factor implicated in the response to hypoxia. Its regulation is governed by HIF-prolyl hydroxylase (HIF-PH), which exhibits oxygen-dependent activity. Notably, it targets key molecules, such as erythropoietin, which elevates oxygen levels in organs by stimulating red blood cell production. The application of HIF-PH inhibitors represents a novel approach to managing renal anemia using hypoxic responses to regulate erythropoietin production. In November 2019, Japan introduced the world’s first HIF-PH inhibitor for treating renal anemia in patients undergoing dialysis. Subsequently, in August 2020, two additional HIF-PH inhibitors received national health insurance listings as treatments for renal anemia in patients with conservative chronic kidney disease. This development is expected to pave the way for the introduction of more HIF-PH inhibitors into clinical practice.

In contrast to conventional injectable erythropoiesis-stimulating agents (ESAs), which exert their effects specifically on the hematopoietic system, these orally administered HIF-PH inhibitors offer systemic effects. Theoretically, they may provide bioprotective benefits by inducing defense mechanisms against hypoxia. However, concerns arise regarding potential side effects arising from the HIF-induced triggering of defense mechanisms in pathological conditions where angiogenesis exacerbates diseases such as cancer and retinal disorders. Nevertheless, the frequency of hypothyroidism induced by HIF-PH inhibitors remained undetermined until a case emerged in Japan in 2021 [1].

In this report, we present two cases of central hypothyroidism resulting from the use of the HIF-PH inhibitor roxadustat in patients with antineutrophil cytoplasmic antibody (ANCA)-associated vasculitis.

## Case presentation

Case 1

**Figure 1:**
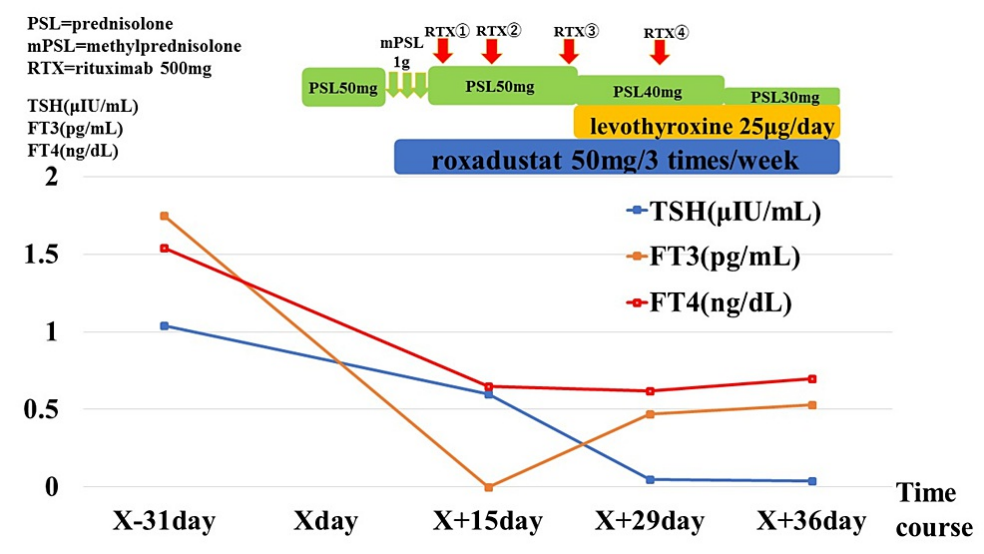
Treatment course and thyroid function in Case 1 On X-31 day, her TSH, FT3, and FT4 levels were within the normal range (TSH 1.04 μIU/mL, FT3 1.75 pg/mL, and FT4 1.54 ng/mL). On X+8 day, roxadustat was introduced to manage renal anemia. By X+15 day, TSH, FT3, and FT4 levels had decreased significantly after administration of roxadustat (TSH 0.605 μIU/mL, <0.39 pg/mL, and 0.65 ng/mL, respectively). Additionally, FT3 and FT4 levels improved with oral levothyroxine use, while roxadustat was continued. However, TSH did not improve. FT3, free triiodothyronine; FT4, free thyroxine; TSH, thyroid-stimulating hormone

A woman in her 80s experienced fever, general malaise, and reduced appetite from X-45 day. She received antibiotic treatment under a local doctor’s care on X-30 day; however, her condition did not improve. Subsequently, on day X, the patient was admitted to the hospital. Her serum urea nitrogen and creatinine levels were elevated to 52 and 1.68 mg/dL (serum urea nitrogen; normal range 8.0-20.0 mg/dL, serum creatinine; normal range 0.61-1.04 mg/dL (male) and 0.47-0.79 mg/dL (female)), respectively, and her myeloperoxidase (MPO)-ANCA level reached 89.4 IU/mL (MPO-ANCA; normal range <3.5 IU/mL). Following admission, a renal biopsy confirmed the diagnosis of ANCA-associated vasculitis, specifically microscopic polyangiitis. She was treated with steroid pulse therapy, prednisolone, and rituximab; however, her renal function continued to deteriorate. A blood test showed that her hemoglobin (Hb) level had dropped to 8-9 g/dL (serum Hb; normal range 11.5-15.0g/dL), and on X+8 day, roxadustat was introduced to manage renal anemia. Her renal function continued to deteriorate, and hemodialysis commenced on X+15 day. Serum Hb stabilized around 10 g/dL after the initiation of roxadustat. She did not originally have thyroid disease, but blood tests performed during hemodialysis revealed central hypothyroidism; her thyroid-stimulating hormone (TSH; normal range 0.35-4.94 μIU/mL), free triiodothyronine (FT3; normal range 1.68-3.67 pg/mL), and free thyroxine (FT4; normal range 0.7-1.48 ng/dL) levels were 0.605 μIU/mL, <0.39 pg/mL, and 0.65 ng/mL, respectively (X-31 day: TSH 1.04 μIU/mL, FT3 1.75 pg/mL, and FT4 1.54 ng/mL (within the normal range)). Tests for TSH receptor, anti-thyroglobulin, and anti-thyroid peroxidase (anti-TPO) antibodies were negative. Other pituitary hormone levels remained unaltered, and the pituitary MRI revealed no discernible neoplastic lesions. Ultrasonography of the thyroid gland revealed no abnormal findings. She presented with general malaise and leg edema, but it was unclear whether this was due to microscopic polyangiitis or central hypothyroidism. Subsequently, the patient consulted an endocrinologist, who initiated low-dose levothyroxine therapy for “central hypothyroidism of unknown cause.” She continued receiving oral roxadustat; routine monitoring indicated gradual improvement in her thyroid function. However, her general condition deteriorated owing to microscopic polyangiitis, ultimately leading to her death on X+49 day. The case progression is displayed in Figure 1.

Case 2

**Figure 2:**
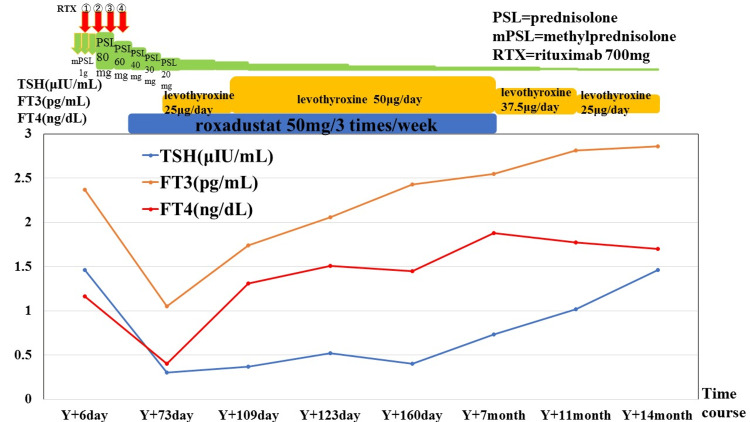
Treatment course and thyroid function in Case 2 On Y+6 day, his thyroid function was within the normal range (TSH: 1.46 μIU/mL, FT3: 2.37 pg/mL, and FT4: 1.16 ng/mL). On Y+28 day, roxadustat was initiated. By Y+73 day, TSH, FT3, and FT4 levels had decreased substantially after administration of roxadustat (TSH 0.30 μIU/mL, FT3 1.05 pg/mL, and FT4 0.40 ng/mL). FT3 and FT4 normalized after the initiation of oral levothyroxine, while roxadustat was continued, and TSH improved mildly. Levothyroxine was tapered off after the end of oral roxadustat, but it was still necessary; TSH showed an increasing trend after the end of roxadustat. FT3, free triiodothyronine; FT4, free thyroxine; TSH, thyroid-stimulating hormone

A man in his 60s with type 2 diabetes mellitus presented with reduced urine output and gross hematuria since Y-40 day. Blood tests revealed a decline in renal function (0.92 mg/dL→3.62 mg/dL) and an increased MPO-ANCA level (9.6 IU/mL). He was referred to our hospital on day Y and admitted on Y+6 day. Elevated serum urea nitrogen and creatinine levels (54 and 5.1 mg/dL, respectively) and an MPO-ANCA level of 18.3 mg/dL were noted. A renal biopsy revealed semilunar formation, leading to the diagnosis of ANCA-associated vasculitis, specifically microscopic polyangiitis. Despite treatment with steroid pulse therapy, prednisolone, and rituximab, his renal function did not improve, and renal anemia persisted. Roxadustat was initiated on Y+28 day. He did not originally have thyroid disease, and his thyroid function on Y+6 day was within the normal range (TSH: 1.46 μIU/mL, FT3: 2.37 pg/mL, and FT4: 1.16 ng/mL). However, on Y+73 day, his thyroid function significantly declined (TSH: 0.30 μIU/mL, FT3: 1.05 pg/mL, and FT4: 0.40 ng/mL). Central hypothyroidism was suspected; however, tests for TSH receptor, anti-thyroglobulin, and anti-TPO antibodies and assessment of other pituitary hormone levels yielded unremarkable results. Pituitary MRI displayed no evident neoplastic lesions, and thyroid gland ultrasonography revealed no irregularities. He was aware of a mild general malaise but was able to carry out his daily activities without problems. It was not clear whether the symptoms were due to microscopic polyangiitis or central hypothyroidism. The patient consulted with an endocrinologist, who initiated low-dose levothyroxine therapy for “central hypothyroidism of an unknown cause.” Roxadustat administration continued, with ongoing thyroid hormone testing revealing a gradual improvement in thyroid function. The patient was transferred to a dialysis clinic near his home for ongoing dialysis treatment, and he ceased taking roxadustat once his Hb level exceeded 12 g/dL on Y+180 day. Subsequently, thyroid function remained stable without exacerbation even after tapering off levothyroxine. The case progression is displayed in Figure 2.

## Discussion

The development of hypothyroidism as a side effect of various medications can be categorized into five classes: (1) inhibition of thyroid hormone synthesis and secretion; (2) inhibition of TSH secretion; (3) acceleration of thyroid hormone metabolism; (4) augmentation of thyroxine-binding globulin; and (5) hindrance of thyroid hormone absorption. Drugs typically associated with hypothyroidism include iodine, lithium carbonate, and dopamine. Central hypothyroidism, as described here, aligns with category (2), characterized by low FT4, low to normal TSH levels, clinical symptoms, and relevant findings.

Central hypothyroidism can be divided into pituitary and hypothalamic hypothyroidism. Distinguishing between the two on a pathophysiological basis is challenging owing to the absence of a well-established measurement system for thyrotropin-releasing hormone (TRH) in the bloodstream. Consequently, they are collectively referred to as central hypothyroidism. Causative factors for central hypothyroidism encompass intracranial-occupying lesions (e.g., pituitary tumors, craniopharyngiomas, and metastatic tumors), vascular etiologies (e.g., Sheehan syndrome and pituitary apoplexy), trauma, radiation therapy, infection, drug-induced triggers, genetic anomalies, and idiopathic origins. Pituitary tumors are the most common cause. The diagnosis of drug-induced hypothyroidism relies on the course of the disease, specifically the temporal relationship between disease onset and medication usage. The decision to discontinue the relevant drug depends on its therapeutic efficacy and the adverse effects associated with discontinuation. In the two cases described, roxadustat-induced central hypothyroidism was not initially suspected. Instead, it was diagnosed as “central hypothyroidism of unknown cause,” and treatment commenced with the administration of low-dose levothyroxine. Previous case reports have indicated that thyroid function improved upon discontinuation of roxadustat and a switch to ESA formulations [1,2]. However, in Case 2, elevated TSH and FT4 levels persisted after discontinuing roxadustat, even after gradual levothyroxine tapering, suggesting roxadustat-induced central hypothyroidism. Case 2 also differs from previous reports in that thyroid function did not normalize promptly after roxadustat discontinuation, requiring long-term thyroid hormone medication administration (for at least one year).

Corticosteroids can also induce hypothyroidism by suppressing TSH secretion via inhibiting TRH gene expression in the hypothalamus. However, prolonged high-dose corticosteroid therapy typically does not result in hypothyroidism. This is likely attributed to the more potent elevation of TSH caused by decreased FT4 and FT3 levels than to the TSH suppression effect of corticosteroids. In cases of corticosteroid-induced hypothyroidism, it is common for TSH to be elevated and FT3 and FT4 to be decreased. In the two patients in this study, TSH, FT3, and FT4 were all highly decreased in a short period of time, making corticosteroid-induced hypothyroidism unlikely. In addition, hypothyroidism due to ANCA-associated vasculitis can result in low levels of circulating T3 hormone due to temporary pathology or stress, a condition called low T3 syndrome. However, TSH and FT4 are often within normal ranges. In the present two cases, TSH, FT3, and FT4 were all highly decreased in a short period of time, making hypothyroidism (low T3 syndrome) due to ANCA-related vasculitis unlikely. Based on the above, we diagnosed the two cases of central hypothyroidism as drug-induced central hypothyroidism caused by roxadustat.

**Figure 3:**
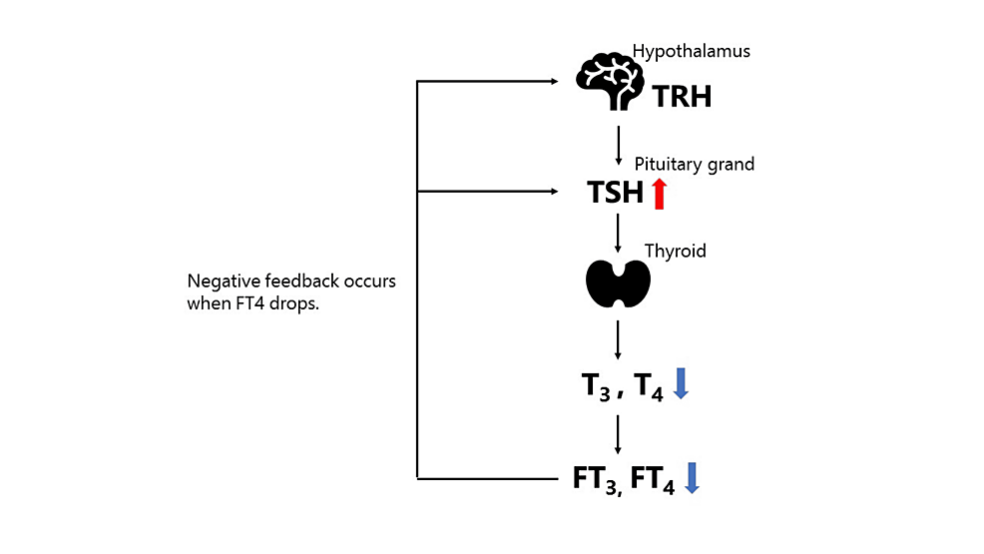
Normal thyroid hormone negative feedback When thyroid hormone levels are insufficient, the negative feedback mechanism of thyroid hormone operates to increase the secretion of TSH, prompting FT3 and FT4 to normalize. FT3, free triiodothyronine; FT4, free thyroxine; TSH, thyroid-stimulating hormone

**Figure 4:**
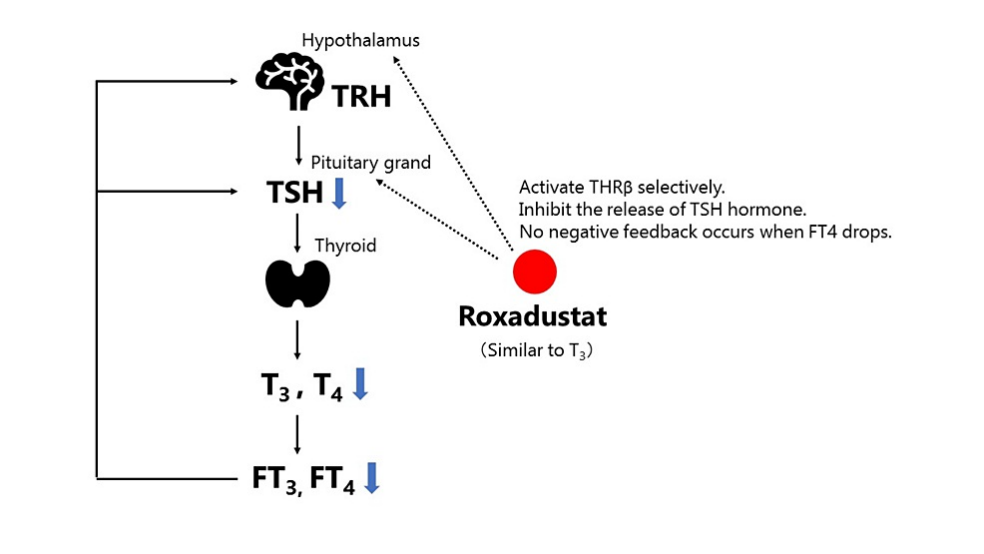
Action mechanism of roxadustat-induced central hypothyroidism Roxadustat shares a structural similarity with T3, allowing it to penetrate the blood-brain barrier and interact with the pituitary thyroid hormone receptor (THRβ). This interaction inhibits the negative feedback loop regulating TSH secretion from the pituitary gland. Consequently, TSH levels decrease, leading to reduced thyroid hormone secretion. As TSH levels fail to normalize, FT3 and FT4 remain low. FT3, free triiodothyronine; FT4, free thyroxine; T3, triiodothyronine; TSH, thyroid-stimulating hormone

The mechanism underlying central hypothyroidism induced by HIF-PH inhibitors has been elucidated as follows: roxadustat, owing to its structural similarity to T3, crosses the blood-brain barrier and interacts with the pituitary thyroid hormone receptor (THRβ) [3,4]. This interaction inhibits the negative feedback on TSH secretion in the pituitary gland (as illustrated in Figure 3 and Figure 4), resulting in reduced TSH levels and decreased thyroid hormone secretion. Renal anemia associated with impaired renal function in ANCA-associated vasculitis is often associated with elevated ferritin levels and impaired iron utilization, rendering HIF-PH inhibitors a common prescription choice. When prescribing the HIF-PH inhibitor roxadustat, regular assessment of thyroid function is imperative, with drug discontinuation considered in the presence of compromised thyroid function. Currently, there are no reports of central hypothyroidism induced by HIF-PH inhibitors other than roxadustat. Hence, a transition to alternative HIF-PH inhibitors or ESA formulations must be considered. Among the two previously reported cases of central hypothyroidism induced by roxadustat, one patient solely switched from roxadustat to an ESA. In contrast, the other shifted from roxadustat to an ESA and increased the levothyroxine dosage, ultimately restoring thyroid function. However, in our cases, both patients demonstrated improved thyroid function upon commencing levothyroxine therapy while continuing roxadustat administration (Table 1). The mechanism underlying central hypothyroidism induced by HIF-PH inhibitors has been clarified. Roxadustat, because of its structural resemblance to T3, crosses the blood-brain barrier and interacts with the pituitary thyroid hormone receptor (THRβ) [3,4]. This interaction inhibits negative feedback on TSH secretion in the pituitary gland (as depicted in Figure 4), resulting in reduced TSH levels and subsequently decreased thyroid hormone secretion. Renal anemia linked to impaired renal function in ANCA-associated vasculitis often exhibits elevated ferritin levels and compromised iron utilization, leading to HIF-PH inhibitors being commonly prescribed. Routine assessment of thyroid function is crucial when prescribing roxadustat, and discontinuation of the drug should be considered if there are signs of compromised thyroid function. To date, no reports of central hypothyroidism induced by HIF-PH inhibitors other than roxadustat have surfaced. Hence, consideration should be given to transitioning to alternative HIF-PH inhibitors or ESA formulations.

**Table 1:** Comparison of past and present cases of roxadustat-induced central thyroid dysfunction Two previously reported cases of central hypothyroidism caused by roxadustat, as well as our two cases, are listed in the table. In all cases, TSH levels decreased markedly after starting roxadustat, and all four patients eventually regained their thyroid function. Of the two previous patients (Tokuyama et al.’s and Ichii et al.’s cases), both had thyroid disease; one patient transitioned from roxadustat to an ESA, and the other patient transitioned from roxadustat to an ESA and increased levothyroxine dose. However, in our patients, both had no thyroid disease; their thyroid function improved with the initiation of levothyroxine while continuing roxadustat. TSH: μIU/mL, FT3: pg/mL, and FT4: ng/mL ESA, erythropoiesis-stimulating agent; FT3, free triiodothyronine; FT4, free thyroxine; TSH, thyroid-stimulating hormone

					Before			After			
Case	Author	Age	Sex	History of thyroid disease	TSH	FT3	FT4	TSH	FT3	FT4	Treatment
1	Tokuyama et al. [1]	85	M	Hypothyroidism due to chronic thyroiditis	2.781	2.5	1	0.038	2	0.8	Switch from roxadustat to ESA
2	Ichii et al. [2]	77	M	Subclinical primary hypothyroidism	NA	NA	NA	0.006	NA	NA	Switch from roxadustat to ESA
3	Omote et al. (our case)	80s	F	N	1.04	1.75	1.54	0.605	<0.39	0.65	Continue roxadustat and start levothyroxine
4	Omote et al. (our case)	60s	M	N	1.46	2.37	1.16	0.3	1.05	0.4	Continue roxadustat and start levothyroxine

Previously reported cases of central hypothyroidism induced by roxadustat involve one patient who switched solely to an ESA and another who transitioned to an ESA while increasing levothyroxine dosage, eventually restoring thyroid function. However, in our cases, both patients exhibited improved thyroid function upon initiating levothyroxine therapy while still on roxadustat (see Table 1). Typically, thyroid function is not evaluated in dialysis patients without known thyroid disease. In our current cases, roxadustat was continued after the onset of hypothyroidism due to the lack of information about roxadustat-induced central hypothyroidism at that time. Essentially, when roxadustat induces central hypothyroidism, it is advisable to discontinue the drug and switch to an ESA preparation or another HIF-PH inhibitor. In our cases, roxadustat was continued alongside levothyroxine.

In the preceding case (1), the patient was initially on levothyroxine for hypothyroidism, and within one month of roxadustat use, TSH decreased from 2.781 to 0.038 μIU/ml, with only a slight decrease in FT3 and FT4. TSH normalized after discontinuing roxadustat for one month. In the previous case (2), the patient was using levothyroxine for hypothyroidism and took roxadustat for four months, resulting in a decrease in TSH to 0.006 μIU/ml with minimal change in FT4. TSH normalized after discontinuing roxadustat for two months. Notably, the present case differs in that the patient had no preexisting thyroid disease and was not initially on levothyroxine. Consequently, the changes in TSH, FT3, and FT4 were distinct from those in previous cases. Particularly, FT4 was notably low due to the absence of levothyroxine.

**Figure 5:**
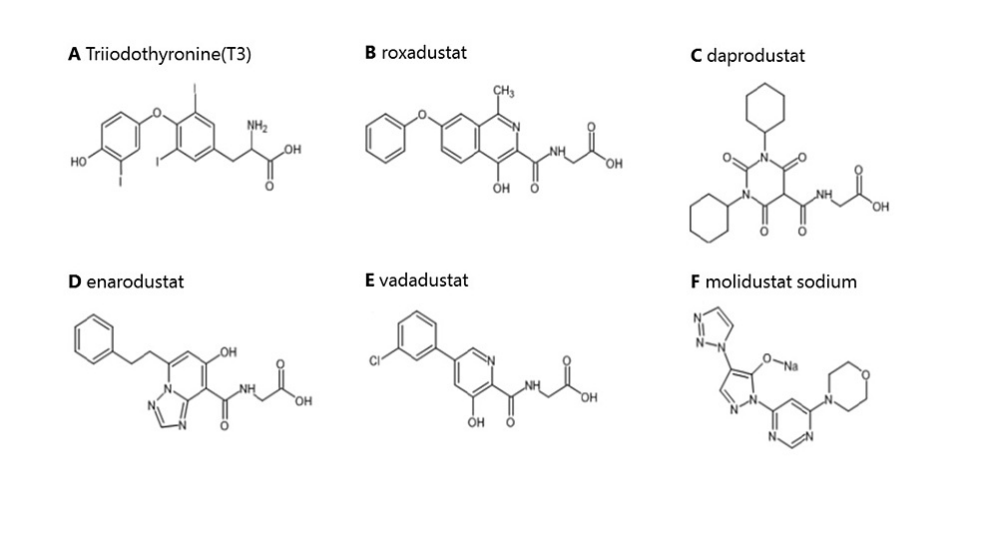
Structural formulas for T3 and currently marketed HIF-PH inhibitors T3 and roxadustat have similar structural formulas and are responsible for central hypothyroidism. The structural formulas of drugs, in addition to roxadustat, which is sold in Japan, are also listed. HIF-PH, hypoxia-inducible factor-prolyl hydroxylase; T3, triiodothyronine

The number of dialysis patients using HIF-PH inhibitors is on the rise, with several HIF-PH inhibitors introduced recently. As of 2024, other HIF-PH inhibitors marketed in Japan besides roxadustat include daprodustat, enarodustat, vadadustat, and molidustat sodium (Figure 5). If the central hypothyroidism caused by roxadustat is due to its structural similarity to T3, it may also occur with other HIF-PH inhibitors. Although our study does not address transitioning from roxadustat to other drugs, future investigations and observations are crucial to bridge this information gap.

## Conclusions

Regular assessment of thyroid function is imperative when administering roxadustat to monitor potential changes in thyroid hormone levels.
